# Arginase 2 Deficiency Prevents Oxidative Stress and Limits Hyperoxia-Induced Retinal Vascular Degeneration

**DOI:** 10.1371/journal.pone.0110604

**Published:** 2014-11-06

**Authors:** Jutamas Suwanpradid, Modesto Rojas, M. Ali Behzadian, R. William Caldwell, Ruth B. Caldwell

**Affiliations:** 1 Vascular Biology Center, Georgia Regents University, Augusta, Georgia, United States of America; 2 Culver Vision Discovery Institute, Georgia Regents University, Augusta, Georgia, United States of America; 3 Department of Pharmacology and Toxicology, Department of Cellular Biology & Anatomy, Georgia Regents University, Augusta, Georgia, United States of America; 4 Charlie Norwood VA Medical Center, Augusta, Georgia, United States of America; Children's Hospital Boston, United States of America

## Abstract

**Background:**

Hyperoxia exposure of premature infants causes obliteration of the immature retinal microvessels, leading to a condition of proliferative vitreoretinal neovascularization termed retinopathy of prematurity (ROP). Previous work has demonstrated that the hyperoxia-induced vascular injury is mediated by dysfunction of endothelial nitric oxide synthase resulting in peroxynitrite formation. This study was undertaken to determine the involvement of the ureahydrolase enzyme arginase in this pathology.

**Methods and Findings:**

Studies were performed using hyperoxia-treated bovine retinal endothelial cells (BRE) and mice with oxygen-induced retinopathy (OIR) as experimental models of ROP. Treatment with the specific arginase inhibitor 2(S)-amino-6-boronohexanoic acid (ABH) prevented hyperoxia-induced apoptosis of BRE cells and reduced vaso-obliteration in the OIR model. Furthermore, deletion of the arginase 2 gene protected against hyperoxia-induced vaso-obliteration, enhanced physiological vascular repair, and reduced retinal neovascularization in the OIR model. Additional deletion of one copy of arginase 1 did not improve the vascular pathology. Analyses of peroxynitrite by quantitation of its biomarker nitrotyrosine, superoxide by dihydroethidium imaging and NO formation by diaminofluoroscein imaging showed that the protective actions of arginase 2 deletion were associated with blockade of superoxide and peroxynitrite formation and normalization of NOS activity.

**Conclusions:**

Our data demonstrate the involvement of arginase activity and arginase 2 expression in hyperoxia-induced vascular injury. Arginase 2 deletion prevents hyperoxia-induced retinal vascular injury by preventing NOS uncoupling resulting in decreased reactive oxygen species formation and increased nitric oxide bioavailability.

## Introduction

Retinopathy of prematurity (ROP) is a major cause of vision loss in premature infants. In the United States, the Centers of Disease Control reports that almost 500,000 babies, one out of every eight, are born prematurely, each year. [1] Many of them develop ROP. The incidence of ROP is inversely proportional to birth weight and ∼50% of infants born weighing less than 1700 g develop ROP. [2] Clinical observations in human infants and studies in animal models indicate that exposure of the immature retinal blood vessels to relative hyperoxia damages the immature retinal capillaries and impairs vascular development. [3] The resulting vascular insufficiency results in a condition of relative hypoxia as development of the retina continues. This up-regulates growth factors, such as vascular endothelial growth factor (VEGF), leading to pathological angiogenesis. [4], [5]

The mechanisms underlying the vascular injury during ROP are not fully understood. However, disruption of amino acid metabolism may be involved. Preterm infants have been shown to have a deficit in L-arginine, which is nutritionally essential for neonatal development. [6] L-arginine is the substrate of both nitric oxide synthase (NOS) and arginase. NOS catalyzes L-arginine to produce NO and L-citrulline, whereas arginase uses L-arginine to produce urea and ornithine. Hepatic urea production is crucial for ammonia detoxification and L-arginine deficiency in preterm infants can cause severe hypoargininemia, which results in hyperammonemia and organ dysfunction. [6] Studies using a mouse model of oxygen-induced retinopathy (OIR) showed that treatment of neonatal mice with supplemental arginine and glutamine prepared as a dipeptide reduced retinal neovascularization and reduced vascular hyperpermeablity following hyperpoxia exposure. [7] Therefore, alterations in L-arginine metabolism may play a role in the microvascular injury.

The products of L-arginine metabolism by NOS and arginase have been strongly implicated in a variety of angiogenic responses. NO can promote angiogenesis and also regulates vascular tone and remodeling. [8]–[12] Ornithine is processed to form L-proline and polyamines, important for collagen synthesis and cell growth, respectively. Thus, products of both enzymes are needed for proper vascular growth and remodeling. However, dysfunction of both enzymes has been implicated in vascular and retinal injury. Our previous studies have shown that hyperoxia induced death of cultured retinal endothelial cells and vaso-obliteration in the immature retina is associated with NOS mediated increases in peroxynitrite formation. [13],[14] Our studies in models of diabetes and oxidative stress-induced vascular disease have shown that elevated arginase can lead to vascular dysfunction and injury by reducing the availability of L-arginine to NOS, causing it to become uncoupled and to form superoxide which reacts with NO to form peroxynitrite. [15], [16] Our studies in the OIR model also have shown involvement of the mitochondrial arginase isoform, arginase 2, in hyperoxia-induced death of retinal neuronal cells. [17], [18] Thus, the overall aim of the present study was to determine whether the arginase pathway is also involved in hyperoxia-induced retinal vascular injury. Here we present data to show involvement of arginase activity and arginase 2 expression in hyperoxia-induced injury of the retinal microvasculature.

## Results

### Hyperoxia-Induced Endothelial Cell Apoptosis

**Figure 1:**
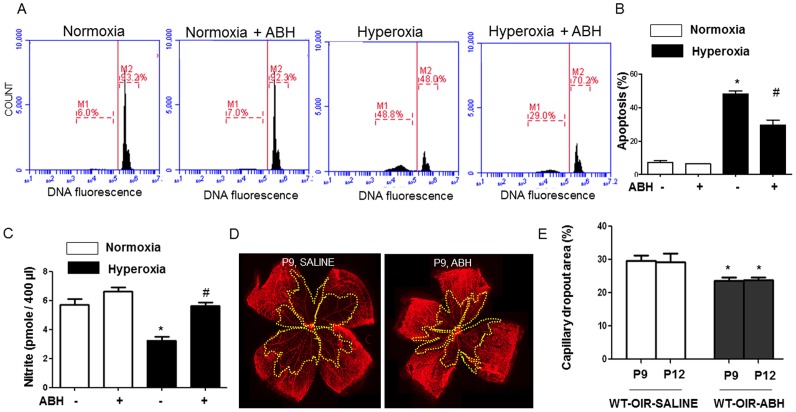
Inhibiting arginase inhibits hyperoxia-induced cell death and preserves nitrite accumulation *in vitro* and vaso-obliteration *in vivo*. For *in vitro* studies, BRECs were treated with hyperoxia (40% O_2_, 5% CO_2_) or normoxia (21% O_2_, 5% CO_2_) with and without ABH (100 µM) for 48 hours. Groups of cells were stained with propidium iodide and the percentage of apoptotic nuclei (hypodiploid, M1) and normal nuclei (diploid, M2) were quantified by flow cytometry (A, B). n = 4–5, * P≤0.05 vs normoxia and hyperoxia treated with ABH, # P≤0.05 vs hyperoxic and normoxic treatment. NO release was detected using chemiluminescence to measure nitrite levels in conditioned media from the treated cells (C. n = 6–9. * P≤0.05 vs normoxia with or without ABH, # P≤0.05 vs hyperoxia without ABH. For *in vivo* analyses, wild type mice were treated with daily i.p. injections of vehicle (saline) or ABH (15 mg/kg) from P7 to P9 or P12. Retinal vessels were visualized by lectin labeling (D) and the area of capillary dropout (yellow) was quantified in fluorescence micrographs using ImageJ (E). n = 7–9, *P≤0.05 vs saline.

We first determined the involvement of arginase activity in hyperoxia-induced endothelial cell death by using a highly selective competitive inhibitor of arginase 2(S)-amino-6-boronohexanoic acid (ABH, 100 µM). [19], [20] BREC treated with or without ABH, were maintained under normoxia (21% O_2_) or hyperoxia (40% O_2_) for 48 hours and stained with propidium iodide. The effect of hyperoxia on apoptosis was evaluated by flow cytometry. This analysis showed that hyperoxia treatment caused a significant increase in numbers of apoptotic cells with hypodiploid nuclei as compared with cells maintained in normoxia (Figure 1A, 1B). Inhibition of arginase activity markedly attenuated hyperoxia-induced apoptosis (Figure 1A, 1B). Next, we quantified the accumulation of nitrite (NO_2_^−^), a product of nitric oxide, in media collected from BREC exposed to normoxia or hyperoxia with and without ABH as described above. Nitrite accumulation was decreased in BREC treated with hyperoxia (Figure 1C). The hyperoxia-induced decrease in nitrite was prevented with ABH treatment (100 µM). This study suggests that inhibition of arginase prevented hyperoxia-induced endothelial cell injury and preserved nitric oxide bioavailability.

### Hyperoxia-Induced Vaso-Obliteration

**Figure 2:**
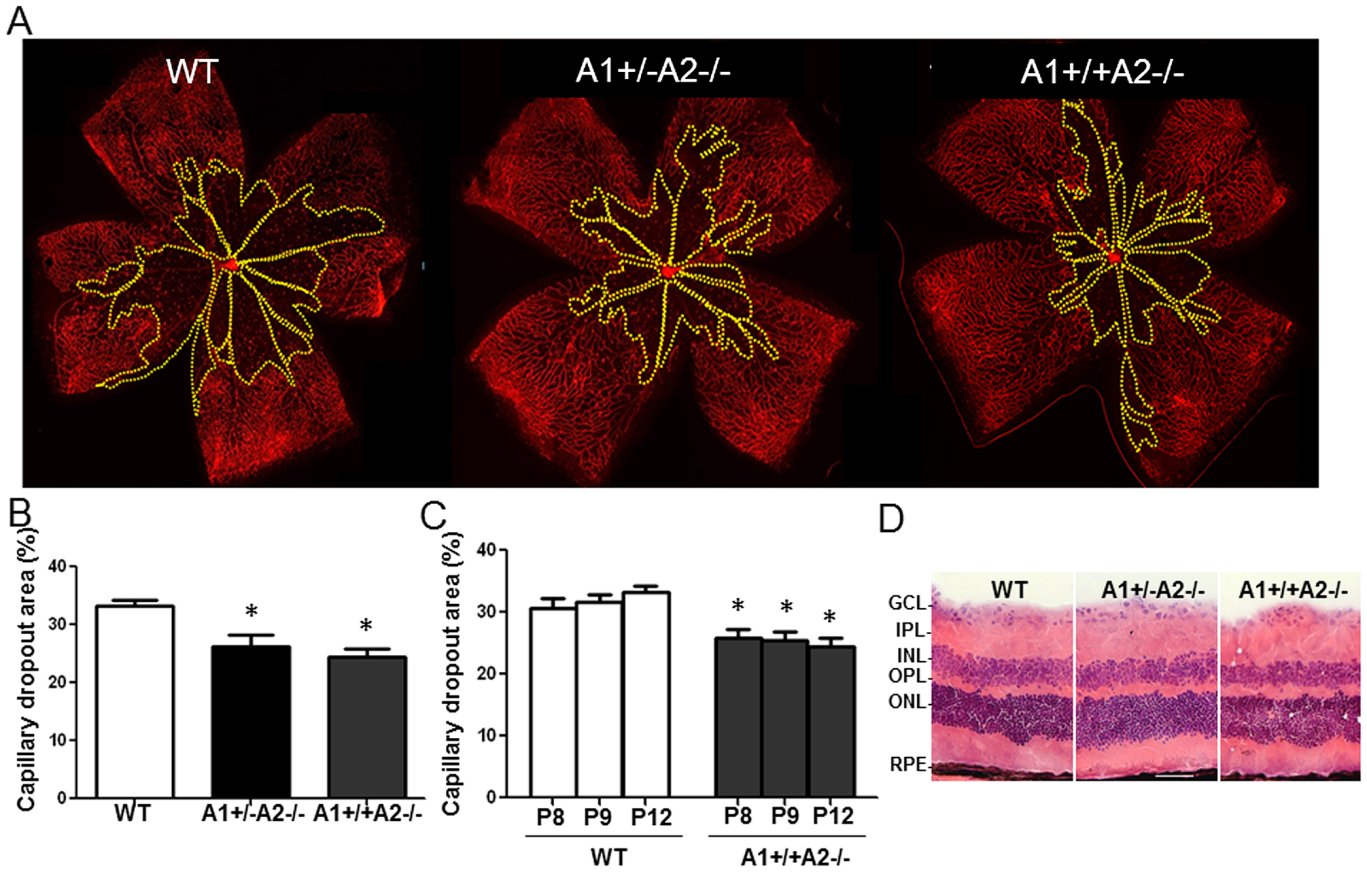
Arginase 2 deletion limits hyperoxia-induced retinal vaso-obliteration. Wild type (WT), arginase 2-deficient mice (A1+/+A2−/−) and arginase-deficient mice lacking one copy of arginase 1 (A1+/−A2−/−) were placed in 70% oxygen on P7 and prepared for analysis on P8, P9 or P12. Retinal vessels were visualized by lectin labeling (A) and the area of capillary dropout (yellow) was quantified in fluorescence micrographs using ImageJ (B, C). n = 5–9, *P≤0.05 vs WT. Images of hematoxylin and eosin stained cryostat sections from adult mice show comparable retinal morphology in WT, A2−/− and A1+/−A2−/− retinas (D, GCL: ganglion cell layer, IPL: inner plexiform layer, INL: inner nuclear layer, OPL: outer plexiform layer, ONL: outer nuclear layer, RPE: retinal pigment epithelium, scale bar  = 50 µm).

In order to evaluate the potential role of arginase activity in hyperoxia-induced retinal vascular injury *in vivo*, we performed experiments in the OIR mouse model. For this, mice were treated with ABH (15 mg/kg/day, ip) beginning on P7 and maintained in 70% oxygen for various times. The isolectin B4-labeled retinas of mice treated with vehicle (saline) and maintained in hyperoxia from P7–12, showed extensive capillary obliteration with a maximal loss at P9 (Figure 1D, 1E). This vessel loss was significantly attenuated in mice treated with ABH, implying a role for arginase in the vascular injury. In order to examine the role of arginase gene expression in this process, we performed similar experiments using mice lacking both copies of the arginase 2 gene alone (A1+/+A2−/−) or in combination with deletion of 1 copy of arginase 1 (A1+/−A2−/−). Deletion of both copies of arginase 1 is lethal by P10 due to hyperammonemia. These data show that the hyperoxia-induced vaso-obliteration was significantly reduced in the A1+/+A2−/− mice as compared with the wild type mice (Figure 2A, 2B). The area of vaso-obliteration in the A1+/−A2−/− mice was not significantly different from that in the mice lacking A2 alone (Figure 2B), suggesting that arginase 1 is not involved in the hyperoxia-induced vaso-obliteration. Time course analysis showed that the protective effect of arginase 2 deletion was evident within 1 day after the onset of hyperoxia and persisted throughout the hyperoxia treatment (Figure 2C). In order to confirm that the retinas of arginase knockout mice develop normally, we also compared retinal morphology in tissue sections prepared from retinas of adult WT, A2−/− and A1+/−A2−/−. As shown in Figure 2D retinal structure qualitatively normal in both lines of mutant mice.

### Vitreo-Retinal Neovascularization during Relative Hypoxia

**Figure 3:**
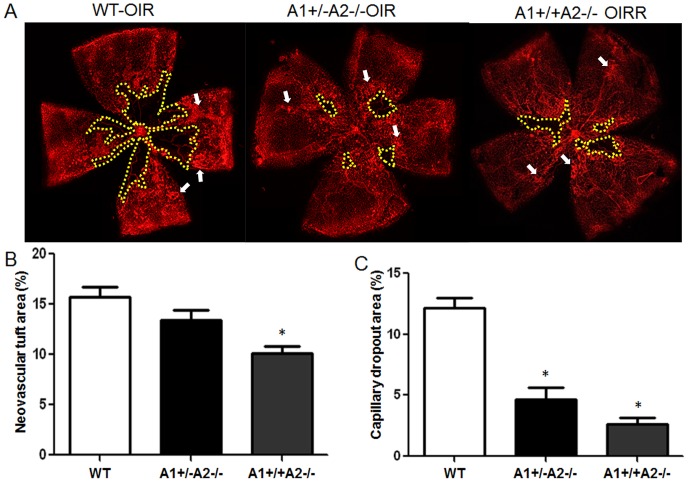
Arginase 2 deletion limits pathological vitreo-retina neovascularization while enhancing intra-retinal neovascularization. Wild type (WT), arginase 2-deficient mice (A1+/+A2−/−) and arginase 2-deficient mice lacking one copy of arginase 1 (A1+/−A2−/−) were maintained in 70% oxygen from P7 to P12, returned to normoxia for 5 days and prepared for analysis on P17. Retinal vessels were visualized by lectin labeling (A) and areas of vitreoretinal neovascular tufts (arrows) and capillary dropout (yellow) were quantified in fluorescence micrographs using ImageJ (B,C). n = 11–13, *P≤0.05 vs WT and A1+/−A2−/− in B and WT in C.

In order to assess involvement of arginase expression in pathological angiogenesis that occurs during the hypoxia phase of OIR, we maintained mice in 70% oxygen from P7 to P12, returned them to normal atmosphere for 5 days and labeled their retinas with isolectin B4 to detect the blood vessels. On P17 extensive vitreo-retinal neovascularization was evident in the wild type mice (Figure 3A). We quantified this pathological angiogenesis by measuring the area occupied by neovascular tufts relative to the total retinal surface area. This analysis revealed that the area of neovascularization in the A1+/+A2−/− retina was significantly reduced as compared to the wild type retina (Figure 3B). Furthermore, in the double mutant mice lacking one copy of arginase 1 as well as both copies of arginase 2, the neovascular tuft formation showed a pattern closer to that in the wild type mice (Figure 3B).

### Intra-Retinal Neovascularization during Relative Hypoxia

We next examined the involvement of arginase expression in physiological retinal angiogenesis/vascular repair during the hypoxia phase of OIR. For this, we measured the capillary-free area relative to the total retinal surface area in the P17 retinas. This analysis showed that vascular repair was significantly enhanced in the A1+/+A2−/− mice as compared with the wild type mice (Figure 3A, 3C). Moreover, the capillary dropout area was slightly increased in the double knockout A1+/−A2−/− as compared with the A1+/+A2−/−mice, suggesting that arginase 1 is not involved in the vascular damage.

### Arginase Expression during Relative Hypoxia

**Figure 4:**
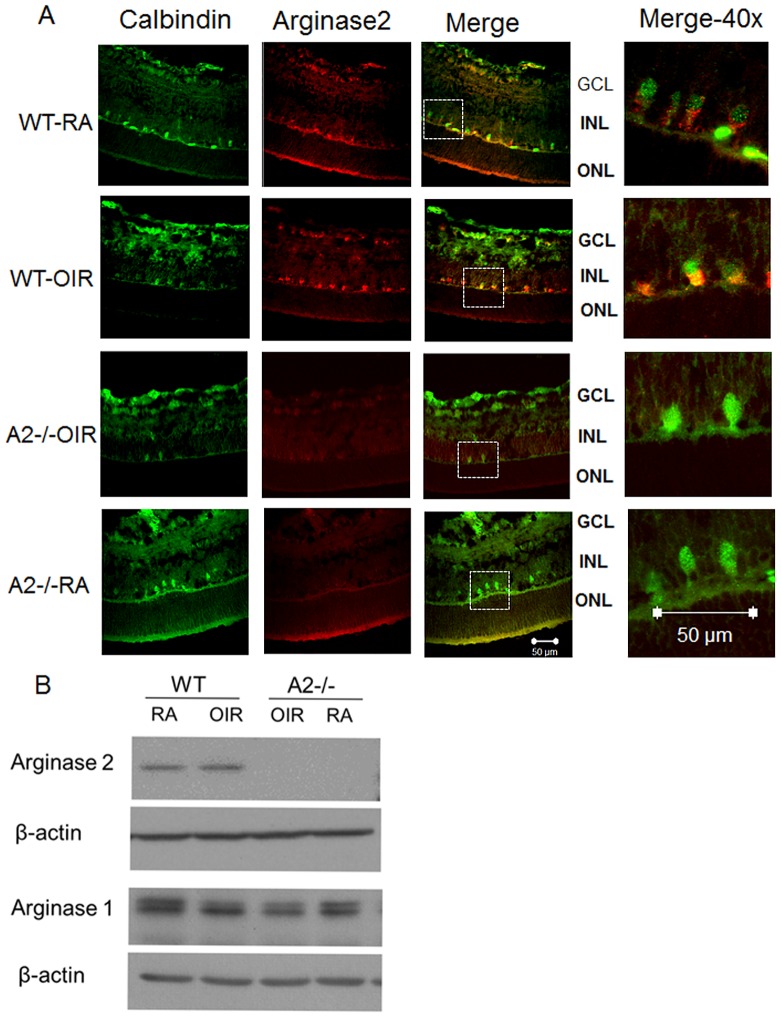
Hyperoxia treatment and Arginase 2 expression in OIR retina. Wild type mice (WT) and mice wild type for arginase 1 and deficient in arginase 2 (A2−/−) were placed in 70% oxygen (OIR) or room air (RA) on P7 and prepared for analysis on after 24 (A) or 48 hr (B). Immunofluorescence imaging (A) of arginase 2 and calbindin in retinal cryosections shows that arginase 2 (red) is highly expressed in horizontal cells (green). Scale bar  = 50 µm, n = 3. Western blot analysis (B) showing arginase 1 and 2 levels following normoxia and hyperoxia treatment, n = 5.

Figure 4 shows the expression of arginase 2 in the retinas of hyperoxia treated mice and their room air (RA) controls. As we have reported previously, immunolocalization analysis showed robust immunoreactivity for arginase 2 in horizontal cells of hyperoxia-treated wild type mice (Figure 4A). [17], [18] Sections prepared from arginase 2 deficient mice were negative, demonstrating specificity of the immunolabeling for arginase 2. Western blot analysis of retinal extracts prepared from wild type mice exposed to hyperoxia for 48 hours showed no change in total levels of arginase 2 as compared with the normoxia controls (Figure 4B). Similarly, western blots prepared after 6, 12, or 24 hours of hyperoxia also showed no change in total levels of arginase 2 as compared with the normoxia controls (data not shown).

### Formation of Peroxynitrite, Superoxide and Nitric Oxide during Hyperoxia

**Figure 5:**
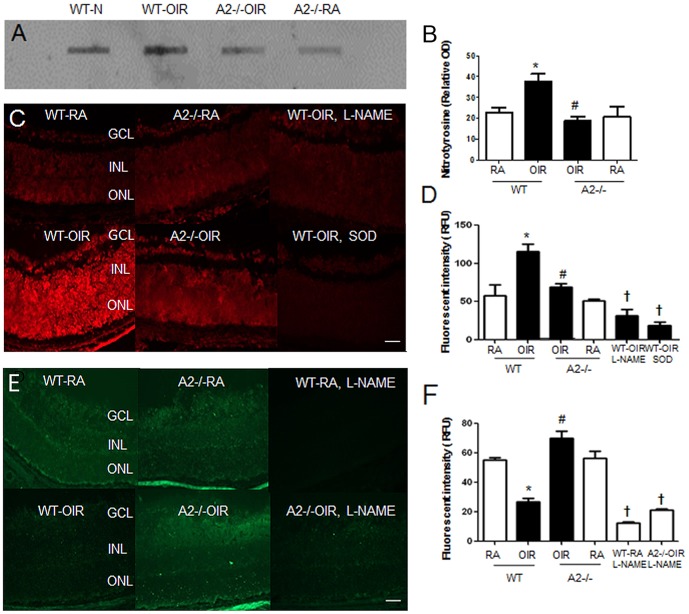
Arginase 2 deletion prevents hyperoxia-induced increases in retinal nitrotyrosine (A) and superoxide (C) levels and preserves NO formation (E). Wild type (WT) and mice wild type for arginase 1 and deficient in arginase 2 (A2−/−) were placed in 70% oxygen (OIR) or room air (RA) on P7 and prepared for analysis on P8. Nitrotyrosine levels in retina samples were detected by slot blot (A) and quantified using ImageJ software (B). n = 6, *P≤0.05 vs WT-RA, # P≤0.05 vs WT-OIR. DHE imaging of superoxide formation was performed using flash frozen retinal sections (C). Results were quantified using Metamorph Imaging System (D). n = 3–5, scale bar  = 50 µm, *P≤0.05 WT-OIR vs all groups; # = P≤0.05 vs WT-OIR. † P<0.05 vs WT-OIR. NO formation in situ was determined by DAF-2-DA fluorescence imaging (E). Results were quantified using Metamorph Imaging System (F). n = 3, scale bar  = 50 µm, * P≤0.05 vs WT-RA, # P≤0.05 vs WT OIR, † P<0.05 vs WT-RA, A2-/-OIR, A2-/-RA.

Previous studies have linked arginase activity to vascular dysfunction and injury by a mechanism involving uncoupling of NOS leading to formation of superoxide which reacts rapidly with NO to produce peroxynitrite. [21] Our previous studies have shown that hyperoxia-induced degeneration of retinal microvascular endothelial cells is associated with increased NOS-dependent peroxynitrite formation. [13], [14] To examine the involvement of arginase 2 in this process, we assayed nitrotyrosine levels in wild type and arginase 2-deficient mice following hyperoxia exposure. While peroxynitrite is short-lived at physiological pH, nitrotysine is a stable end product of the peroxynitrite reaction with tyrosine residues in proteins. Thus, nitrotyrosine serves as a biomarker for peroxynitrite. [22]–[24] Our analysis demonstrated a two fold increase in levels of nitrated proteins in retinas of hyperoxia treated wild type mice as compared with the normoxia controls (Figure 5A, 5B). By contrast, nitrotyrosine levels remained low in the hyperoxia-treated arginase 2 deficient mice, suggesting involvement of arginase 2 expression in the hyperoxia-induced increase in peroxynitrite formation.

To examine the involvement of NOS uncoupling in this process, we used DHE imaging to determine the effects of the hyperoxia treatment on NOS-dependent formation of superoxide in flash frozen retina sections pre-treated with and without the NOS inhibitor L-NAME. This analysis showed a 2 fold increase in DHE fluorescence intensity in the retinas of the hyperoxia-treated wild type mice compared with the normoxia controls (Figure 5C, 5D). Since DHE can be oxidized by other ROS beside superoxide, [25] Control experiments were performed using SOD which catalyzes superoxide to hydrogen peroxide. The SOD treatment markedly blunted the DHE fluorescence, indicating involvement of superoxide in the reaction. The hyperoxia-induced increase in DHE fluorescence was also markedly reduced by pre-treatment of the retina sections with the NOS inhibitor L-NAME, This implies that NOS activity is an important contributor to superoxide production. Moreover, the hyperoxia-induced increase in DHE staining was suppressed in the arginase 2 deficient mice. Taken together, these data suggest that arginase 2 has an important role in hyperoxia-induced superoxide formation by a mechanism involving NOS uncoupling.

We further examined the role of arginase 2 expression in hyperoxia-induced NOS dysfunction by analyzing NO formation using DAF-FM imaging of retina sections from wild type and arginase 2-deficient mice pre-treated with and without L-NAME. The NO signal was markedly reduced in sections from hyperoxia-treated wild type mice as compared with the other groups (Figure 5E, 5F). By contrast the NO signal was preserved in the retinas of the hyperoxia-treated arginase 2-deficient mice. L-NAME treatment abrogated the DAF-FM signal in both wild type and arginase 2 deficient retinas, demonstrating specificity of the reaction for NO. These data indicate that arginase 2 deletion preserves normal NOS function and NO formation in the OIR mouse model.

## Discussion

Hyperoxia-induced obliteration of newly formed blood vessels in the immature retina has been well established as a primary mediator of retinopathy of prematurity. [3] Our previous studies *in vivo* and *in vitro* have shown involvement of NOS mediated increases in peroxynitrite formation in hyperoxia-induced vaso-obliteration and endothelial cell death. [14], [26] Now we demonstrate involvement of the arginase enzyme in this pathology. Studies in retinal endothelial cells showed that treatment with the specific arginase inhibitor ABH blocked hyperoxia-induced apoptosis and preserved NO availability. *In vivo* experiments using the mouse OIR model showed that inhibition of arginase activity or deletion of the arginase 2 gene significantly reduced hyperoxia-induced vaso-obliteration. Deletion of arginase 2 also limited pathological vitreoretinal neovascularization while enhancing vascular repair during the relative hypoxia phase of OIR. Interestingly, deletion of one copy of the arginase 1 gene in combination with both copies of arginase 2 was less effective in reducing vitreoretinal neovascularization. This suggests that arginase 2 is the predominant isoform involved in the vascular injury. Immunoblot analyses of the peroxynitrite biomarker nitrotyrosine showed that deletion of the arginase 2 gene also limited peroxynitrite formation. Our studies further indicated that arginase 2 expression is involved in hyperoxia-induced superoxide formation by a mechanism requiring activity of NOS. Moreover, NO formation by NOS was suppressed in retinas of hyperoxia-treated mice and the hyperoxia-induced suppression of NO formation was prevented in arginase 2 deficient mice. Taken together, these data indicate that hyperoxia induced vascular injury is mediated by arginase 2-induced uncoupling of NOS.

Interestingly, pathological neovascularization was slightly increased in mice lacking 1 copy of arginase as well as both copies of arginase 2 as compared with the mice lacking both copies of arginase 2 alone. Therefore, we performed additional studies to determine the impact of arginase 1 deletion on vascular regrowth into the area of vaso-obliteration following hyperoxia treatment. The data show that deletion of 1 copy of arginase impairs vascular repair during the hypoxic phase of the OIR model (manuscript in preparation). The mechanisms controlling expression and function of arginase 1 and 2 in the retina are as yet unknown. However, studies using human lung endothelial cells have shown that HIF-2 regulates arginase 2 expression and activity during hyperoxia treatment. [27] In addition, ROS-induced upregulation and activation of endothelial arginase 1 is regulated by AP1 mediated activation of the Rho A/ROCK system leading to p38 MAPK activation. [28], [29] In models of artherosclerosis, liver X receptors (LXRs) were found to regulate macrophage arginase 1 [30] while arginase 2 in endothelial cells is regulated by epigenetic mechanisms involving activity of histone deacetylase 2. [31] In the immune system, arginase 1 in macrophage can be regulated by PTEN pathway [32].

This study is the first we know of to show that inhibiting the arginase enzyme or deleting the arginase 2 gene limits hyperoxia-induced injury of the immature retinal vessels. Interestingly, a previous study reported that higher levels of arginase 2 mRNA and an apparent decrease in iNOS activity were associated with decreases in neuronal cell injury during the hypoxia phase of OIR in mice that lack the TNFα gene, implying a neuroprotective role for arginase 2 in OIR. [33] However, our previous studies have shown that deletion of arginase 2 prevents hyperoxia-induced death of retinal neurons during both hyperoxia and relative hypoxia phases of OIR. [17], [18] The neuronal cell death was found to be mediated by a process involving hyperoxia-induced increases in polyamine oxidation.^18^ Studies are now in progress to examine the involvement of alterations in polyamine metabolism in the retinal vascular injury during OIR. Further study is required to reconcile this discrepancy between the effects of arginase 2 deletion in limiting neuronal and vascular injury as shown in our current and previous studies and the association between upregulation of arginase 2 and decreased neuronal injury as shown in the TNF-α deficient mice.

Our data showing decreases in superoxide and peroxynitrite along with increases in NO formation in the arginase 2 deficient retina strongly indicate involvement of arginase 2 mediated uncoupling of NOS in the hyperoxia-induced vascular injury during OIR. One limitation of our results is that we were unable to detect any change in the total amount of arginase 2 protein in the OIR retinas. The lack of an effect of OIR on arginase 2 protein expression can be explained by the fact that arginase 2 is localized mainly in mitochondria and expression levels are low in most cell types. As shown in Figure 4, arginase 2 is strongly expressed in horizontal cells which are few in number and restricted a single layer at the outer border of the inner nuclear layer. The arginase 2 signal appears high in the horizontal cells of the OIR retina as compared with the room air controls. However, our previous work has shown that horizontal cells undergo degeneration in the OIR retina as indicated by TUNEL labeling within calbindin-positive horizontal cells and decreases in number of calbindin-positive cells. By contrast the horizontal cell death is limited in the arginase 2 knockout mice. [18] Thus, the hyperoxia-induced increase in arginase 2 expression may be offset by decreases in the number of arginase 2-positive cells. It is possible that arginase 2 activation is involved in hyperoxia-induced horizontal cell death. The role of retinal horizontal cells in the control of retinal vascular survival and function is not yet known. However, the horizontal cells are distributed in a network across the entire retina and their processes are in contact with the photoreceptors, bipolar cells and amacrine cells as well as with the outermost layer of retinal vessels. [34], [35] They are known to modulate signaling between photoreceptors and bipolar cells. [36] An important function of horizontal cells is sending inhibition signals to enhance spatial contrast of image. [36] Therefore, it is likely that loss of horizontal cells leads to poor vision outcome in ROP patient. Our previous work has shown that arginase 2 deletion reduces OIR-induced impairment of visual function as shown by electroretinography. [17] Thus the horizontal cells are ideally positioned to regulate microvascular function in relation to neuronal activity. The specific functions of these neurovascular interactions are as yet unknown. Further study is needed to elucidate the role of the horizontal cell alterations in the vascular and neuronal injury during ischemic retinopathy.

Due to the limited resolution of confocal microscope imaging and the small volume of vascular tissue in the 10 µm frozen sections used for our immunolocalization studies, we were unable to clearly localize arginase 2 within the retinal vascular cells. Our previous analyses have demonstrated arginase 2 expression in retinal vessels isolated from mice as well as in cultured BRECs. [37] More studies will be needed to determine the specific effects of hyperoxia exposure on arginase 2 expression and activity in vascular endothelial cells.

Our finding that deletion of arginase 2 in mice prevents hyperoxia-induced obliteration of the retinal vasculature while preventing NOS-dependent ROS formation and preserving NO availability is consistent with previous studies showing involvement of excessive arginase 1 in hyperoxia-induced vascular injury in the developing lung [38] and excessive arginase 2 in vascular dyfunction associated with atherosclerosis and aging. [39], [40], [41] Moreover, in other retinal disease models, including acute inflammation and diabetes, excessive arginase 1 has been shown to dysregulate endothelial function through interfering with NOS function. [8], [42], [43] These differences suggest that differences in vascular maturity, target tissue and/or specific disease-associated triggers determine the arginase isoform involved in vascular injury.

Further work is needed to define the specific mechanisms by which arginase 2 deletion limits ROS formation, preserves NOS function and protects the retinal vessels during hyperoxia treatment. Several studies have shown that arginase 2 up-regulation in endothelial cells causes vascular dysfunction and injury via NOS uncoupling. [39], [40], [44] It is possible that hyperoxia-induced activation of arginase 2 triggers NOS uncoupling by depleting L-arginine, oxidizing tetrahydrobiopterin (BH4) and/or increasing endogenous methylarginine, all of which can lead to reduction in NO availability and buildup of ROS. [45] A previous study using the OIR mouse model indicated that eNOS over-expression reduced BH4 leading to vascular injury during hyperoxia. [46] Arginase 2 activation can also increase oxidative stress in multiple cell types by increasing polyamine oxidation due to activation of the ornithine/polyamine pathway. Our previous studies have shown that arginase 2 deletion limits hyperoxia-induced increases in oxidative stress and reduces injury and death of the retinal neurons by a mechanism involving suppression of hyperoxia-induced activation of polyamine oxidase. [17], [18], [37] Arginase 2 deletion also limits microglial activation in our OIR model (manuscript in preparation). Studies are in progress to better understand the relationship between the neuronal and vascular cell injury and the role of the arginase pathway in this pathology.

## Conclusions

In summary, clinical studies have shown low levels of L-arginine and L-glutamine in infants with ROP, [6] and treatment with an arginyl-glutamine dipeptide supplement was found to lessen vascular damage in the OIR mouse model. [7] Reduction in L-arginine appears to involve up-regulation of arginase activity. Excessive arginase activity is not only involved in L-arginine deficiency, but it is apparently associated with enhanced polyamine production along with polyamine oxidation which is toxic to cells. Our studies suggest that limiting arginase activity, particularly arginase 2, is a promising means of treating oxidative stress-related retinopathy.

## Materials and Methods

### Cell Culture Analysis and Nitric Oxide Measurement

Primary cultures of BREC were prepared as described previously. [47] Cells from *passages 5–8* were used in all experiments. Cells were maintained in M199 supplemented with 10% FBS, 10% CS-C complete medium, 2 mM glutamine, 100 U/ml penicillin, and 100 µg/ml streptomycin at 37°C in a humidified CO_2_ incubator. For analysis of hyperoxia effects on apoptosis, BREC were grown to 70% confluence and switched to serum-free medium. The next day they were placed in a hyperoxic (40% O_2_, 5% CO_2_) or normoxic (21% O_2_, 5% CO_2_) environment for 48 h as described by us previous. [14] Cells were treated with or without ABH (100 µM). To quantify apoptosis, cells were trypsinized, washed with phosphate buffer saline (PBS) and resuspended in propidium iodide (PI) solution (0.1% triton, 0.1% sodium citrate, and 50 mg/L PI, Sigma-Aldrich, St. Louis, MO) for one hour. [48] A minimum of 30,000 cells/sample were analyzed on a flow cytometer (BD Accuri C6, San Jose, CA) using a 488-nm laser line for excitation and >600 nm for detecting the emission. The percentage of apoptotic (M1) and normal nuclei (M2) was obtained by analysis of the DNA histogram. For analysis of NO release, conditioned media (200 µl) was collected from cells treated as described for the apoptosis study was treated with ethanol (200 µl) to remove proteins and refluxed in sodium iodide/glacial acetic acid to convert NO_2_^−^ to NO. NO was measured via NO-specific chemiluminescence after reaction with ozone using a Sievers NO analyzer (GE Analytical Instruments, Boulder, CO). [49] Net NO_2_^−^ release from cells treated with ABH or vehicle under normoxic or hyperoxic condition was calculated after subtracting NO_2_^−^ content in the base media.

### Ethics Statement

All procedures with animals were carried out in strict accordance with the recommendations in the Guide for the Care and Use of Laboratory Animals of the National Institutes of Health. The protocol was approved by the Committee on the Ethics of Animal Experiments of Georgia Regents University Animal Care and Use Committee (Animal Welfare Assurance no. A3307-01). Euthanasia was performed by thoracotomy under avertin (2, 2, 2 tribromoethanol, Sigma) anesthesia, and all efforts were made to minimize suffering.

### Treatment of Animals

Experiments were performed with C57BL/6J wild-type mice and mice deficient in arginase 1 or/and arginase 2 (A1+/−A2−/−, A1+/−A2+/+, A1+/+A2−/−). These lines were developed by Drs. S. D. Cederbaum [50] and W. E. O'Brien [51] and were provided by Dr. Cederbaum with the permission of Dr. O'Brien. Some mice were treated with the arginase inhibitor 2(S)-amino- 6-boronohexanoic acid (ABH, kindly provided by Dr. Dan Berkowitz). Room temperature was maintained at 20°C, and illumination was provided by standard fluorescent lighting on a 12-hour light–dark cycle.

Oxygen-induced retinopathy was induced in newborn mice according to the protocol of Smith, et al. with some adjustment. [52] On postnatal day 7 (P7), mice were placed along with their dams into 70% oxygen for up to 5 days, after which they were transferred back to room air. The 70% oxygen concentration was used because the mutant mice do not tolerate 75% oxygen treatment. The animals were sacrificed at various times. Following deep anesthesia with Avertin (250 mg/kg, ip), mice were euthanized by thoracotomy and the eyeballs were taken out and fixed in 4% paraformaldehyde (PFA) overnight. Retina flat-mounts were dissected and labeled with biotinylated *Griffonia simplicifolia* isolectin B4 (Vector Laboratories). Retinas were viewed with fluorescence microscopy (Axiophot, Zeiss, Chester, VA) and the images captured in digital format (Spot System; Diagnostic Instruments, Sterling Heights, MI). Central capillary dropout Image-J software [52].

### Histology and Morphology of Retina Cross Sections

Retinal morphology was studied on retinal cryostat sections from WT, A2-/-, and A1+/−A2−/− OIR adult mice. Sections (10 µm) containing the optic nerve were collected 20 um apart from one another, and stained with hematoxylin and eosin (H&E, Fisher Scientific).

### Immunofluorescence

Eyes were removed, fixed in 4% paraformaldehyde, washed in PBS and retinas were isolated and cryoprotected. Cryostat sections (10 um) were permeabilized in 1% Triton (20 min) and blocked in 10% normal goat serum containing 1% BSA (1 h). Sections were then incubated overnight in primary antibodies (4°C): (rabbit polyclonal anti-arginase 2, 1∶250, Santa Cruz Biotechnology, Dallas, TX; mouse monoclonal anti-calbindin, 1∶200, Sigma-Aldrich, St. Louis, MO) followed by reaction with fluorescein or Texas red conjugated secondary antibodies (Life Technologies, Grand Island, NY), washed in PBS and mounted with Vectashield (Vector Laboratories, Burlingame, CA).

### Western Blotting

Retinas were homogenized in a modified RIPA buffer [20 mmol/L Tris-HCl (pH 7.4), 2.5 mmol/L ethylenediamine tetraacetic acid, 50 mmol/L NaF, 10 mmol/L Na4P2O7, 1% TritonX-100, 0.1% sodium dodecyl sulfate, 1% sodium deoxycholate, 1 mmol/L phenylmethyl sulfonyl fluoride] and 10 µg protein samples were separated by 10% sodium dodecyl sulfate-polyacrylamide gel electrophoresis, transferred to nitrocellulose membrane, and reacted with antibodies (chicken polyclonal anti-arginase 1, 1∶20,000, kindly provided by Dr. Sidney Morris; rabbit polyclonal anti Arginase 2 antibody (1∶250, Santa Cruz Biotechnology, Dallas, TX) followed by horseradish peroxidase-linked secondary antibody and enhanced chemiluminescence (Amersham Pharmacia, San Francisco, CA). Membranes were stripped and reprobed for β-actin to demonstrate equal loading.

### Nitrotyrosine Slot Blot

Relative amounts of proteins nitrated on tyrosine were measured by use of slot-blot techniques. Assay lysate was transferred onto nitrocellulose membrane using a slot-blot microfiltration unit (Bio-Rad Laboratories, Hercules, CA). Nitrotyrosine was detected by use of a mouse monoclonal anti-nitrotyrosine antibody (Cayman Chemical, Ann Harper, MI) followed by followed by horseradish peroxidase-conjugated secondary antibody (1∶2000, GE Healthcare Bio-Sciences, Pittsburgh, PA). Immunoreactive proteins were detected using the enhanced chemiluminescence (ECL) system (GE Healthcare Bio-Sciences, Pittsburgh, PA). Relative levels of nitrotyrosine immunoreactivity were determined by Image J software.

### Dihydroethidium Fluorescence Imaging of Superoxide

Superoxide production was evaluated in retinal frozen sections by the dihydroethidium (DHE) method, as described previously. [43] Briefly, frozen sections were preincubated in NADPH (100 µM) with or without PEG-SOD (400 U/mL) or NOS inhibitor L-NG-nitroarginine methyl ester (L-NAME, 100 µmol, 1 mM) for 20 minutes, followed by reaction with DHE (2 µM) for 20 minutes at 37°C. DHE images from serial sections treated with or without inhibitors were obtained using a fluorescence microscope (AxioVision; Carl Zeiss, Thornwood, NY). DHE was excited at 470 nm with an emission spectrum of 610 nm. The images were analyzed for reaction intensity using computer assisted morphometry (Metamorph Image System; Molecular Devices, Downingtown, PA).

### Diaminofluorescein Diacetate Imaging of Nitric Oxide

NO production was evaluated in retinal frozen sections using 4,5-diaminofluorescein diacetate (DAF-2 DA, EMD Millipore, Billerica, MA). [43] Briefly, frozen sections were pre-incubated with HEPES or NOS inhibitor L-NG-nitroarginine methyl ester (L-NAME; 100 µmol (1 mM) for 20 minutes, followed by reaction with DAF-2 DA for 20 minutes at 37°C. Slices were then washed 3 times for total of 15 min with fresh HEPES. NO images from serial sections treated with or without inhibitors were obtained using a fluorescence microscope (AxioVision). DAF was excited at 488 nm with an emission spectrum at 515 nm. The images were analyzed for reaction intensity (Metamorph Image System).

### Statistical Analysis

All data were summarized as means ±SEM. Statistical methods to were used to compare treatment groups and determine significance of observed differences in all experiments. In each case the data were reviewed to see how well they fit the assumptions of the tests. In most cases the comparisons were between multiple groups and the overall differences were analyzed by ANOVA followed by Tukey multiple comparison to evaluate group differences. P values <0.05 were taken as significant.
